# Patient Involvement in Care, Psychosocial Outcomes, and Quality of Life in Hypertrophic Cardiomyopathy: A Pilot Study

**DOI:** 10.1016/j.cjco.2023.11.002

**Published:** 2023-11-04

**Authors:** Braeden Hill, Nicholas Grubic, Kiera Liblik, Amer M. Johri

**Affiliations:** a Department of Medicine, Division of Cardiology, Cardiovascular Imaging Network at Queen’s University, Kingston, Ontario, Canada; b Dalla Lana School of Public Health, University of Toronto, Toronto, Ontario, Canada

## Abstract

This cross-sectional study evaluated the impact of patient involvement in care (PIC) on psychosocial outcomes and health-related quality of life (HRQoL) in patients with hypertrophic cardiomyopathy (HCM) (n = 34). Patients with low-to-moderate PIC were older than those with high PIC (66.8 years vs 57.3 years; *P* = 0.04). PIC was negatively correlated with depressive symptoms (*r* = –0.39; *P* = 0.02) and positively correlated with heart-focused attention (*r* = 0.39; *P* = 0.02). No significant correlations were observed between PIC and HRQoL. Greater PIC was associated with reduced depressive symptoms but increased cardiac anxiety. Future studies should investigate the relationship between PIC and HRQoL in a larger cohort.

## Résumé

Cette étude transversale visait à évaluer l’effet de la participation du patient à ses soins sur les issues psychosociales et la qualité de vie liée à la santé chez les patients atteints de cardiomyopathie hypertrophique (CMH) (n = 34). Les patients qui participaient peu ou modérément à leurs soins étaient plus âgés que ceux qui y participaient activement (66,8 ans *vs* 57,3 ans; *p* = 0,04). Il y a une corrélation négative entre la participation du patient aux soins et les symptômes dépressifs (*r* = -0,39; *p* = 0,02) et une corrélation positive entre la participation aux soins et l’attention portée au cœur (*r* = 0,39; *p* = 0,02). Aucune corrélation notable n’a été observée entre la participation du patient à ses soins et la qualité de vie liée à la santé. Une grande participation du patient à ses soins a été associée à une réduction des symptômes dépressifs, mais à une anxiété cardiaque accrue. D’autres études sont nécessaires pour examiner la relation entre la participation du patient à ses soins et la qualité de vie liée à la santé au sein d’une cohorte plus importante.

Hypertrophic cardiomyopathy (HCM) is a congenital heart disease characterized by pathologic hypertrophy of the left ventricle (LV). HCM has historically posed a significant global health burden as a cause of cardiac arrhythmias, sudden cardiac death (SCD), and heart failure (HF), although with contemporary management, patients may have relatively normal longevity and low disease-related mortality.^1^ Despite a favourable prognosis, patients with HCM report poorer psychosocial outcomes and health-related quality of life (HRQoL) than the general population.^2^^,^^3^ This may be attributable to disease symptomology, risk of adverse events, and restrictive measures of care made through paternalistic decision making.^2^, ^3^, ^4^ The emergence of shared decision making (SDM) has shifted clinical risk stratification toward a dependence upon both cardiologists’ clinical judgement as well as the preferences and values of informed patients.^5^ Despite support of SDM in recent HCM reviews and guidelines,^5^^,^^6^ there is a paucity of evidence describing the impact of patient involvement in care (PIC) in this population. As such, this study aimed to evaluate the relationship of PIC with psychosocial and HRQoL outcomes in patients with HCM.

## Methods

We conducted a cross-sectional, single-centre survey study of HCM outpatients. This study was approved by the Queen’s University Health Sciences and Affiliated Teaching Hospitals Research Ethics Board (#6036973, DMED-2678-22). All participants provided informed consent before entering the study.

Eligible patients with HCM were identified by review of medical records and prospectively recruited after outpatient appointments with a cardiologist at the Kingston Health Sciences Center (KHSC) Adult Congenital Heart Disease (ACHD) clinic. Patients were included if they were ≥ 18 years of age; diagnosed with HCM based on genetic or echocardiographic findings; and able and willing to provide informed consent. Patients were excluded if they were enrolled in the study previously; diagnosis query based on clinical suspicion of HCM; and if they were unable or unwilling to provide informed consent.

Participants were administered a baseline questionnaire to ascertain sociodemographic and clinical characteristics (Supplemental Appendix S1). PIC, psychosocial, and HRQoL outcomes were evaluated using psychometric scales validated in cardiovascular and outpatient populations. The Modified-Perceived Involvement in Care Scale (M-PICS) is used to evaluate patients’ perceptions of patient-health care provider communication with respect to the establishment of good interpersonal relationships, the promotion of information exchange, and the facilitation of patient involvement.^7^ The M-PICS consists of 20 items rated on a 5-point scale of 1 to 5, with a minimum score of 20, maximum score of 100, and higher scores indicating greater perceived involvement in care.^7^ Scores were stratified from 20 to 46, 47 to 73, and 74 to 100 to describe low-, moderate-, and high-perceived involvement in care, respectively. The Hospital Anxiety and Depression Scale (HADS) is used in the clinical setting to delineate severity of depression and anxiety symptoms without confounding somatic symptoms.^8^^,^^9^ The Cardiac Anxiety Questionnaire (CAQ) measures anxiety related to cardiovascular health and associated behaviours such as checking pulse often or avoiding physical activity because of fear of increasing heart rate.^10^ The 12-item Kansas City Cardiomyopathy Questionnaire (KCCQ-12) is a condensed iteration of the original instrument,^11^ that evaluates HRQoL in patients with cardiomyopathies and has been validated in clinical trials of HCM patients.^12^ A detailed description of the psychometric survey instruments applied in this study is provided in Supplemental Appendix S2.

Participant characteristics were stratified by low to moderate (20-73) and high (74-100) M-PICS summary score and reported using descriptive statistics. The distribution of categorical variables was reported as frequencies/percentages, whereas the distribution of continuous variables was reported as means/standard deviations. Demographic characteristics between M-PICS groups were compared using Fisher’s exact test for categorical variables and the Mann-Whitney U test for continuous variables. Pearson’s correlation was used to assess the degree of correlation between M-PICS and psychosocial and HRQoL outcomes. Linear correlations between continuous M-PICS scores and continuous scores on each domain of the HADS, CAQ, and KCCQ-12 were evaluated and displayed graphically. Correlations were reported using Pearson’s correlation coefficient (*r*), wherein greater absolute value of coefficients (ranging from 0-1) indicates stronger correlation and negative coefficients indicate an inverse correlation among variables. Coefficients ranging from 0 to 0.09, 0.10 to 0.39, 0.40 to 0.69, 0.70 to 0.89, and 0.90 to 1.00 were defined as negligible, weak, moderate, strong, and very strong correlation, respectively.^13^ Statistical analyses were performed using SAS version 9.4 software (SAS Institute, Cary, NC), and statistical significance was inferred as *P* < 0.05.

## Results

The demographic and clinical characteristics of the study population are presented in Table 1. A total of 41 HCM outpatients of the KHSC ACHD clinic were eligible to participate in this study; 34 patients consented and completed the study questionnaire. Eighteen patients (52.9%) were classified as low-to-moderately involved in their care (M-PICS summary score ≤ 73), whereas 16 (47.1%) were classified as highly involved in their care (M-PICS summary score ≥ 74). The mean age of the study population was 62.4 (± 17.5), which differed between low-to-moderate and high M-PICS groups (66.8 ± 16.9 vs 57.3 ± 17.3; *P* = 0.04). Most patients were prescribed medication for their HCM (76.5%). Patients who reported low-to-moderate involvement in care were prescribed medication less often than those who reported high involvement in care (61.1% vs 93.4%; *P* = 0.04).

**Table 1:** Demographic and clinical characteristics of the study population

Variable	Total (n = 34)	Low-to-moderate M-PICS score (≤ 73) (n = 18)	High M-PICS score (≥ 74) (n = 16)	*P* value
Demographic characteristics				
Age∗	62.4 (± 17.5)	66.8 (± 16.9)	57.3 (± 17.3)	0.04†
Woman gender identity	16 (47.1%)	9 (50%)	7 (43.8%)	0.74
Non-White racial identity	5 (14.7%)	1 (5.6%)	4 (25%)	0.16
High school education or lower	12 (35.3%)	9 (50%)	3 (18.8%)	0.08
Unemployed or retired	21 (61.8%)	14 (77.8%)	7 (43.8%)	0.08
< $40,000 CAD household income	11 (32.4%)	6 (33.3%)	5 (31.3%)	1.00
Single marital status‡	20 (58.8%)	12 (66.7%)	8 (50%)	0.49
Smoker	13 (38.2%)	8 (44.4%)	5 (31.3%)	0.50
Comorbidities				
Previous diagnosis depression	11 (32.4%)	7 (38.9%)	4 (25%)	0.48
Previous diagnosis anxiety	6 (17.6%)	3 (16.7%)	3 (18.8%)	1.00
High cholesterol	13 (38.2%)	8 (44.4%)	5 (31.3%)	0.50
Diabetes	6 (17.7%)	2 (11.1%)	4 (25%)	0.39
Hypertension	18 (52.9%)	9 (50%)	9 (56.3%)	0.74
Body mass index > 30 (obese)	12 (35.3%)	5 (27.8%)	7 (43.8%)	0.48
Clinical characteristics				
Prescribed HCM medication	26 (76.5%)	11 (61.1%)	15 (93.4%)	0.04†
Septal myectomy or septal ablation history	6 (17.6%)	3 (16.7%)	3 (18.8%)	1.00
ICD history	8 (23.5%)	3 (16.7%)	5 (31.3%)	0.43
Less than yearly cardiology visit	9 (26.5%)	6 (33.3%)	3 (18.8%)	0.45
Physically inactive/sedentary	18 (52.9%)	11 (61.1%)	7 (43.8%)	0.49
Athlete (recreational or competitive)	7 (20.6%)	5 (27.8%)	2 (12.5%)	0.41

CAD, Canadian dollars; HCM, hypertrophic cardiomyopathy; ICD, implantable cardioverter defibrillator; M-PICS, Modified-Perceived Involvement in Care Scale.

∗ Mean (± SD)

† Statistically significant *P* value.

‡ Separated, divorced, widowed, never married.

**Figure 1:**
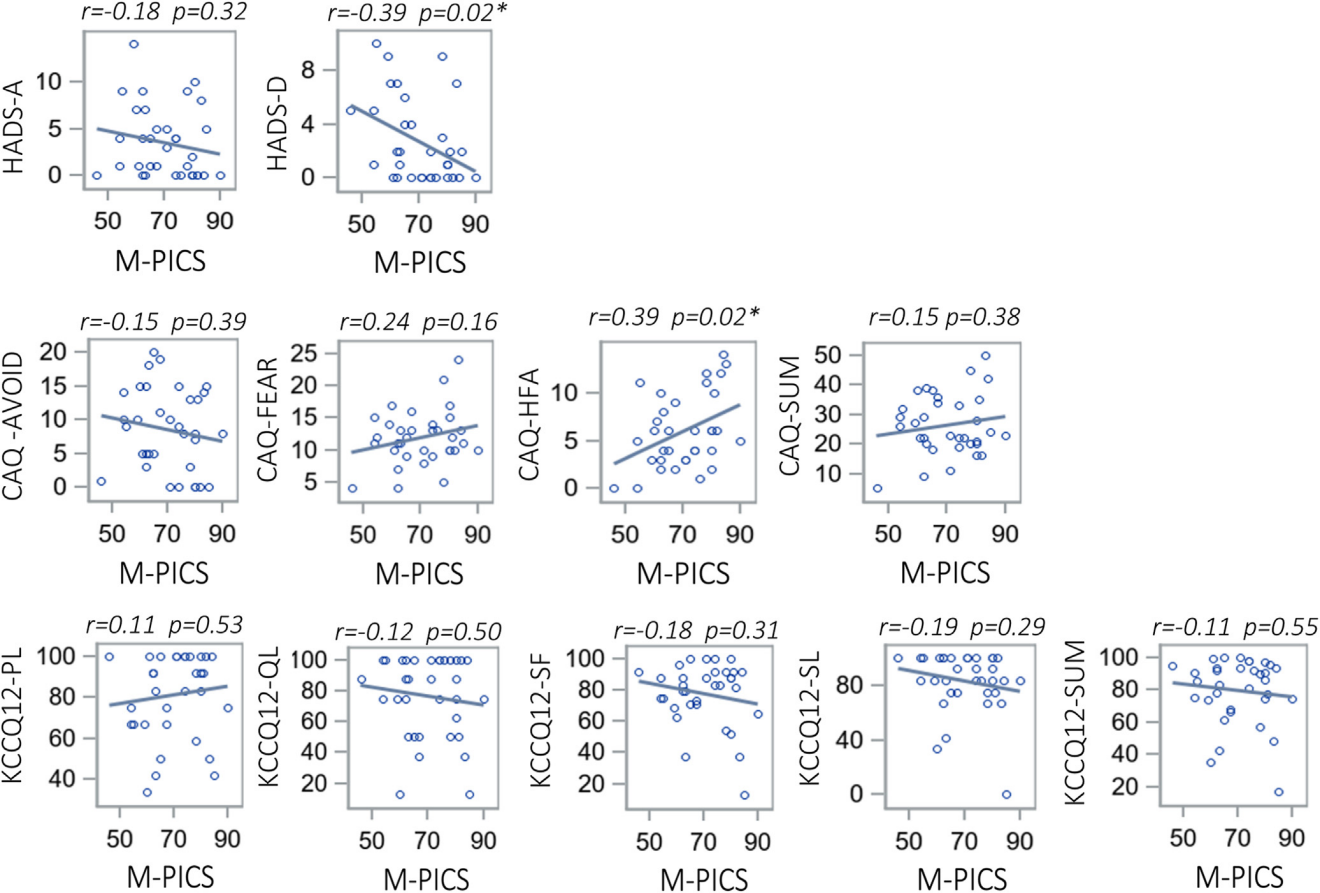
Pearson correlation plots depicting linear correlations between continuous Modified-Perceived Involvement in Care Scale (M-PICS) summary score and psychosocial and health-related quality-of-life scale scores. CAQ, Cardiac Anxiety Questionnaire; CAQ-AVOID, Avoidance Subscale of CAQ; CAQ-FEAR, Fear subscale of CAQ; CAQ-HFA, Heart-Focused Attention subscale of CAQ; CAQ-SUM, Summary score of CAQ; HADS-A, Anxiety subsection of Hospital Anxiety and Depression scale; HADS-D, Depression subsection of Hospital Anxiety and Depression scale; KCCQ12, 12-item Kansas City Cardiomyopathy Questionnaire; KCCQ12-PL, Physical Limitation subscale of KCCQ12; KCCQ12-QL, Quality-of-Life subscale of KCCQ12; KCCQ12-SF, Symptom Frequency subscale of KCCQ12; KCCQ12-SL, Social Limitation subscale of KCCQ12; KCCQ12-SUM, Summary score of KCCQ12. **∗**Statistically significant correlation.

The results of the Pearson correlation analysis between PIC and psychosocial and HRQoL outcomes are presented in Figure 1 and Table 2. M-PICS summary score had a significant weak negative correlation with depressive symptoms as indicated by HADS-Depression (*r* = –0.39; *P* = 0.02). A significant weak positive correlation was found between M-PICS and the heart-focused attention subscale of the CAQ (*r* = 0.39; *P* = 0.02). None of the correlations between the M-PICS and KCCQ-12 subscales achieved statistical significance. The median and range scores for psychosocial and HRQoL outcomes among the study sample are presented in Supplemental Appendix S3.

**Table 2:** Pearson’s correlation analysis of M-PICS with psychosocial and HRQoL outcomes

Psychometric scale	M-PICS correlation (*r*)	*P* value
Psychosocial outcomes		
HADS Anxiety subscale (HADS-A)	–0.18	0.32
HADS Depression subscale (HADS-D)	–0.39	0.02∗
CAQ Fear subscale (CAQ-FEAR)	0.24	0.16
CAQ Avoidance subscale (CAQ-AVOID)	–0.15	0.39
CAQ Heart-Focused Attention subscale (CAQ-HFA)	0.39	0.02∗
CAQ Summary score (CAQ-SUM)	0.15	0.38
HRQoL outcomes		
KCCQ-12 Physical Limitation subscale (KCCQ12-PL)	0.11	0.53
KCCQ-12 Symptom Frequency subscale (KCCQ12-SF)	–0.18	0.31
KCCQ-12 Quality-of-Life subscale (KCCQ12-QL)	–0.12	0.50
KCCQ-12 Social Limitation subscale (KCCQ12-SL)	–0.19	0.29
KCCQ-12 Summary score (KCCQ12-SUM)	–0.11	0.55

HRQoL, Health-Related Quality-of-Life; KCCQ-12, 12-item Kansas City Cardiomyopathy Questionnaire; M-PICS, Modified Perceived Involvement in Care Scale.

∗ Statistically significant *P* value. *r* = 0-0.10 indicates negligible correlation; *r* = 0.10-0.39 indicates weak correlation; *r* = 0.40-0.69 indicates moderate correlation; *r* = 0.70-0.89 indicates strong correlation; *r* = 0.90-1.00 indicates very strong correlation. Negative (–) indicates inverse correlation.

## Discussion

This is the first study to investigate the relationship of PIC with psychosocial and HRQoL outcomes in patients with HCM. A relatively equal proportion of patients were low-to-moderately (52.9%) and highly involved in care (47.1%), suggesting that disparities in PIC exist in HCM outpatient clinics. Highly involved patients were younger (57.3 ± 17.3 vs 66.8 ± 16.9; *P* = 0.04), indicating that younger patients with HCM may take more active engagement during appointments, whereas older patients remain passive, which has been corroborated by previous studies in breast cancer treatment consultations^14^ and patients postmyocardial infarction.^15^ A greater proportion of patients prescribed HCM medication were highly involved in care (93.4% vs 61.1%; *P* = 0.04). This observation may be attributable to the fact that patients prescribed medication are typically more symptomatic and also may have an enhanced perception of the potential consequences of their disease following initiation of treatment.^16^

Previous studies investigating psychosocial outcomes in patients with HCM suggest that they experience poorer psychosocial well being as indicated by the HADS.^4^^,^^17^^,^^18^ Notably, our study found that M-PICS was weakly negatively correlated with HADS-Depression (*r* = –0.39; *P* = 0.02), suggesting that greater PIC may help reduce the burden of depressive symptoms in patients with HCM or that managing patients’ depressive symptoms may facilitate PIC. Previous research in depressive primary care patients similarly found that greater PIC significantly increased the probability of depression resolution,^19^ whereas depressed patients report more positive perceptions of clinical care during highly patient-centred visits.^20^ M-PICS was positively correlated with most domains of cardiac anxiety indicated by the CAQ, with the weak positive correlation between heart-focused attention and PIC reaching statistical significance (*r* = 0.39; *P* = 0.02). This may suggest that greater involvement in care makes patients with HCM more likely to demonstrate symptoms of cardiac anxiety or that anxious patients may be motivated to maintain greater involvement in care.^10^ We hypothesize that this could be attributable to patients with HCM highly involved in care being more aware of the risks of their condition (ie, SCD) and the precipitating factors of cardiac events (ie, dyspnea, tachycardia, myocardial ischemia), although because of the limitations of our cross-sectional study design, it is unclear as to whether this is a result of greater involvement in care leading to increased anxiety or vice versa.

Previous research has also demonstrated that patients with HCM report impaired HRQoL because of factors related to the physical and social implications of their conditions.^2^^,^^4^^,^^17^^,^^18^ Our results related to HRQoL were less conclusive, with no correlations between M-PICS and KCCQ-12 scales reaching statistical significance. Similar research investigating PIC and HRQoL in patients with chronic illnesses have found conflicting results, with some studies suggesting there is no relationship between PIC and HRQoL,^21^^,^^22^ whereas others suggest greater PIC improves HRQoL,^23^, ^24^, ^25^ although there is little evidence to suggest a negative association.^26^ Future research should aim to further delineate the relationship between PIC and HRQoL to address this gap in knowledge.

### Limitations

There are several limitations to this study. First, the study population was limited in sample size and demographic diversity because of its recruitment from a small outpatient clinic serving a region geographically distant from large multicultural hubs. This limited our ability to detect significant differences between groups and variables of interest. Second, because of the pilot nature of this study, participation involved only baseline data collection, with no opportunity to collect follow-up data to investigate changes in PIC or health outcomes. Moreover, patients could not be recruited upon their index visit, as there were too few patients to capture a sample of patients new to HCM care. Third, given the cross-sectional design of this study, temporality between PIC and psychosocial outcomes cannot be assumed, which may prevent causal inferences. There is a need for future longitudinal studies to further evaluate this relationship. With the correlative analysis of this cross-sectional study, there is potential for reverse causality, as it remains uncertain whether PIC enacts influence on psychosocial symptoms or whether psychosocial symptoms are more likely to influence levels of PIC.

Future research should aim to determine the impact of PIC on psychosocial and HRQoL outcomes in patients with HCM while addressing the limitations of the current study. More robust prospective studies with larger samples, recruitment from multiple centres, and longitudinal data collection at multiple points in time may generate statistically significant results that can build upon the preliminary evidence provided in this pilot study.

## Conclusions

Active involvement in care was associated with improved psychosocial outcomes among patients with HCM, particularly with respect to general depression. However, health care providers should be aware that greater involvement in care may be associated with a greater likelihood of developing symptoms of cardiac anxiety and that older patients may be more likely to remain passively involved in their clinical management. As the field of HCM care evolves, this study should serve as a pilot for larger prospective investigations of the implications of PIC on psychosocial and HRQoL outcomes in the context of HCM, informing the development of improved clinical recommendations that will guide the integration of SDM and novel therapeutics into practice.
