# Supplementary material for: Patient Involvement in Care, Psychosocial Outcomes, and Quality of Life in Hypertrophic Cardiomyopathy: A Pilot Study
Source: CJC Open. 2023 Nov 4;6(3):539–43. doi: 10.1016/j.cjco.2023.11.002 (PMC10980899; doi:10.1016/j.cjco.2023.11.002)
Supplement: Supplemental Appendix [file mmc1.pdf]

SUPPLEMENTARY MATERIAL

Patient involvement in care, psychosocial outcomes, and quality of life in hypertrophic cardiomyopathy: a pilot study

Hill B, Grubic N, Liblik K, & Johri AM (2023)

Supplemental Appendix S1. Demographic and clinical characteristics questionnaire

HIRA Study **Baseline Worksheet** Participant ID: _______________

Date: _____________ Ethnicity: ___________________
Age: _____ Racial Identity:_______________________

Baseline Information *(Questions adapted from GENESIS PRAXY Gender Questionnaire – Pelletier 2015)*

Gender Identity	____________________________
Education level (check all that apply):	□ No degree, certificate, or diploma □ High School/GED<br>□ Technical/Vocational Program □ Some College/University<br>□ College Degree □ Undergraduate Degree<br>□ Graduate Degree
Employment Status:	□ Part-Time □ Full-Time □ Unemployed □ Retired<br>□ Homemaker □ Student □ Leave of Absence
Current Job (if applicable):	___________________________________
Household Income Before Taxes:	□ Less than $9,999 □ $10,000 - $14,999<br>□ $15,000 - $19,999 □ $20,000 - $29,999<br>□ $30,000 - $39,999 □ $40,000 - $49,999<br>□ $50,000 - $59,999 □ $60,000 - $69,999<br>□ $70,000 - $79,999 □ $80,000 - $89,999<br>□ $90,000 - $99,999 □ $100,000 - $149,999 □ $150,000+
Marital Status:	□ Never Married □ Separated □ Divorced<br>□ Widowed/Widower □ Married □ Common Law
What best describes your living situation:	□ House Owned by Self/Partner<br>□ House Rented by Self/Partner<br>□ Live with Friends/Family<br>□ No Permanent Residence<br>□ Subsidized Housing<br>□ Hospice/Long-term Care
Smoking (Tobacco):	□ Yes (Currently Smoker) □ No (Have Smoked)<br>□ No (Have Never Smoked)<br><br>**If you are currently smoking cigarettes or have a history of smoking:**<br>______ Cigarettes per Day **OR** ______ Packs per Day<br>*If you no longer smoke, fill in based on the average amount per day during the time you were smoking.*<br>**How long have you been smoking/did you smoke?** ____ Years
Alcohol drinks per week:	_____ per Week (if none, write 0)
Have you been treated for **or** diagnosed with substance use disorder (commonly known as drug addiction):	□ Yes □ No<br>If yes, when? ____________________________________
Have you been treated for or diagnosed with depression:	□ Yes □ No<br>If yes, when? ____________________________________
Have you been treated for or diagnosed with anxiety:	□ Yes □ No<br>If yes, when? ____________________________________
Do you have high cholesterol?	□ Yes (taking medication) □ Yes (not taking medication)<br>□ No
Do you have diabetes?	□ Yes (taking medication) □ Yes (not taking medication)<br>□ No
Do you have hypertension?	□ Yes (taking medication) □ Yes (not taking medication)<br>□ No

What is your height?	_____________
What is your weight?	_____________
**Hypertrophic Cardiomyopathy (HCM) information**	
In what year was your HCM diagnosis:	________________________________
How is/was your HCM managed (check all that apply):	□ Lifestyle modification<br>□ Medication<br>□ Septal myectomy<br>□ Septal ablation<br>□ Implantable cardioverter-defibrillator (ICD)<br>□ Other: ___________________________
Are you working part-time/unemployed/on long-term sick leave/retired from work as a result of your HCM:	□ Yes □ No □ Do not know<br><br>**If you are no longer working full-time as a result of your HCM, for how long have you been working part-time/unemployed/on long-term sick leave/retired from work:**___________________________________
How many days have you taken off work in the last 3 months due to your HCM:	□ None □ 1-5 □ 6-10 □ 11-15 □ 16-20 □ 21 or more
Do you have a family history of HCM:	□ Yes □ No □ Do not know
Do you have a family history of sudden cardiac death:	□ Yes □ No □ Do not know
Have you had any sudden cardiac events related to your HCM:	□ Yes □ No<br>If yes, what? _______________________________
How frequently do you discuss your HCM with your cardiologist/primary care provider (on average):	□ At least once a month □ At least every 3 months<br>□ At least every 6 months □ At least once a year<br>□ Less frequently than once a year
How much moderate- to high-intensity physical exercise do you engage in per week (on average):	□ None □ 1-3 hours □ 4-6 hours □ 7 hours or more
What is your athletic status:	□ None (not engaged in sport)<br>□ Recreational athlete (engage in sport for leisure)<br>□ Competitive athlete (engage in sport in organized competition but not professionally)<br>□ Elite athlete (engage in sport in professional or collegiate leagues)
To what extent have you been restricted from physical activity:	□ No restriction<br>□ Some restriction (i.e., I have only been restricted from engaging in some physical activities)<br>□ Significant restriction (i.e., I have been restricted from engaging in most physical activities)
How do you feel about the extent your activity restriction:	□ Unhappy/upset □ Neutral □ Happy/content □ Not restricted
Do you feel like your physician fairly considered your opinion when prescribing exercise restriction:	□ Yes □ No □ Unsure □ Not restricted

How many medications do you take for your HCM:	□ None □ 1 □ 2 □ 3 □ 4 or more
Do you feel like your physician fairly considered your opinion when prescribing HCM medication(s):	□ Yes □ No □ Unsure □ Not prescribed HCM medication
HCM medication history (from electronic medical records):	____
HCM diagnostics history (from electronic medical records) (check all that apply):	□ Echocardiogram □ ECG □ Cardiac MRI □ Stress test<br>□ Genetic testing

Supplemental Appendix S2. Detailed description of study survey instruments

Modified Perceived Involvement in Care Scale

The M-PICS is an adaptation of the original perceived involvement in care scale (PICS) used to evaluate patient perceptions of their involvement in and quality of care,[1] with modifications made to enhance its application to chronic medical conditions.[2] M-PICS measures patient involvement in care by evaluating perceived healthcare provider-patient communication during medical visits in an inpatient or outpatient setting.[2] The M-PICS consists of 20 items rated on a five-point scale of 1-5, with a minimum score of 20 and maximum score of 100. Higher scores indicate greater perceived involvement in care, with scores from 20-46, 47-73, and 74-100 describing low, moderate, and high perceived involvement in care, respectively. The M-PICS has been validated as a consistent measure of PIC in the context of chronic medical conditions including breast cancer and chronic pain, and in outpatient populations,[1–4] although has not been directly validated in HCM patients. To account for this, the scale was minorly adapted for the purposes of this study by modifying its terminology to be representative of a cardiology clinic setting. To account for the low sample size of our pilot study, we combined the stratifications of low (n=1) and moderate (n=17) PIC indicated by M-PICS to create a low-moderate PIC grouping, to allow for detection of relevant statistical differences between the low-moderate (n=18) and high (n=16) PIC groups.

Hospital Anxiety and Depression Scale

The HADS is used in the clinical setting as it specifically aims to delineate severity of depression and anxiety symptoms without confounding somatic symptoms that may be present due to underlying illness.[5,6] The HADS is composed of 14 items rated on a three-point scale of 1-3, divided into a seven-item depression subscale (HADS-D) and seven-item anxiety subscale

(HADS-A). Using the score cut-off of eight on the anxiety and depression subscales (of a maximum of 21) confers a 93.7% sensitivity for HADS-A and 84.6% sensitivity for HADS-D, in addition to a 72.6% specificity for HADS-A and a 90.3% specificity for HADS-D.[5,6] HADS has been validated in patients with cardiac conditions including HCM.[7–9]

Cardiac Anxiety Questionnaire

The CAQ is specifically used to measure anxiety related to cardiovascular health as well as associated behaviours such as checking pulse often or avoiding physical activity due to fear of increasing heart rate. The CAQ has 18 items rated on a five-point scale of 0-4, with higher scores indicating greater cardiac anxiety.[10] The CAQ is divided into three subscales describing heart-related fear (CAQ-FEAR), avoidant behaviour (CAQ-AVOID), and heart-focused attention (CAQ-HFA). In previous studies, the median CAQ score was successfully used as a CAQ cut-off (24 of 72), and any higher score was indicative of cardiac anxiety.[11–14] Validation studies demonstrate that the CAQ has high internal consistency and is a reliable instrument to identify cardiac anxiety in cardiovascular patients including those with HCM.[12–16]

Kansas City Cardiomyopathy Questionnaire

The KCCQ evaluates functional health status/HRQoL in patients with cardiomyopathies and has been validated and applied in large-scale clinical trials of HCM patients.[17–21] The KCCQ-12 is a condensed iteration designed to make the KCCQ more feasible to integrate into clinical care, while preserving the validity of the original instrument.[17,22] The KCCQ-12 consists of 12 items scored on five-, six-, and seven-point scales. It is divided into four subscales evaluating the physical limitations (KCCQ12-PL), symptom frequency (KCCQ12-SF), quality of life implications (KCCQ12-QL), and social limitations of their condition (KCCQ12-SL), and a summary score describing overall HRQoL (KCCQ12-SUM).[22] Scores are scaled from 0-100, with

greater scores indicating better HRQoL, and overall summary scores <25, 25-49, 50-74, and 75-100 indicating very poor, poor, fair, and good HRQoL, respectively.

Supplemental Appendix S3. Psychosocial and quality of life outcome scores across study sample (n=34)

Psychometric Scale	Median	Range
**Psychosocial Outcomes**		
HADS Anxiety subscale (HADS-A)	3	0–14
HADS Depression subscale (HADS-D)	2	0–10
HADS Summary Score (HADS-SUM)	5	0–23
CAQ Fear subscale (CAQ-FEAR)	12	4–24
CAQ Avoidance subscale (CAQ-AVOID)	9	0–20
CAQ Heart-Focused Attention subscale (CAQ-HFA)	6	0–14
CAQ Summary Score (CAQ-SUM)	24	5–50
**HRQoL Outcomes**		
KCCQ-12 Physical Limitation subscale (KCCQ12-PL)	92	33–100
KCCQ-12 Symptom Frequency subscale (KCCQ12-SF)	83	13–100
KCCQ-12 Quality of Life subscale (KCCQ12-QL)	88	13–100
KCCQ-12 Social Limitation subscale (KCCQ12-SL)	83	0–100
KCCQ-12 Summary Score (KCCQ12-SUM)	86	17–100

CAQ = Cardiac Anxiety Questionnaire; HADS = Hospital Anxiety Depression Scale; KCCQ-12 = 12-item Kansas City Cardiomyopathy Questionnaire. The HADS summary score is scored out of 42, with subscales scored from 0-21 where greater scores indicate greater depression/anxiety. The CAQ summary score is scored out of 72, with the FEAR subscale scored from 0-32 and the AVOID and HFA subscales scored from 0-20, where greater scores indicate greater cardiac anxiety. The KCCQ-12 summary score and subscales are scored and scaled from 0-100, where greater scores indicate greater health-related quality of life.

References

1. Lerman CE, Brody DS, Caputo GC, Smith DG, Lazaro CG, Wolfson HG. Patients' Perceived Involvement in Care Scale: relationship to attitudes about illness and medical care. *J Gen Intern Med*. 1990;5(1):29-33. doi:10.1007/BF02602306
2. Smith MY, Winkel G, Egert J, Diaz-Wionczek M, DuHamel KN. Patient-physician communication in the context of persistent pain: validation of a modified version of the patients' Perceived Involvement in Care Scale. *J Pain Symptom Manage*. 2006;32(1):71-81. doi:10.1016/J.JPAINSYMMAN.2006.01.007
3. Jonsdottir T, Jonsdottir H, Gunnarsdottir S. Validation of the patients' perceived involvement in care scale among patients with chronic pain. *Scand J Caring Sci*. 2013;27(3):740-749. doi:10.1111/J.1471-6712.2012.01066.X
4. Jacobsen R, MØldrup C, Christrup L, SjØgren P, Hansen OB. The Danish version of the questionnaire on pain communication: preliminary validation in cancer patients. *Acta Anaesthesiol Scand*. 2009;53(6):807-815. doi:10.1111/J.1399-6576.2009.01959.X
5. Herrmann C. International experiences with the hospital anxiety and depression scale - A review of validation data and clinical results. *J Psychosom Res*. 1997;42(1):17-41. doi:10.1016/S0022-3999(96)00216-4
6. Zigmond AS, Snaith RP. The hospital anxiety and depression scale. *Acta Psychiatr Scand*. 1983;67(6):361-370. doi:10.1111/J.1600-0447.1983.TB09716.X
7. Poole NA, Morgan JF. Validity and reliability of the Hospital Anxiety and Depression Scale in a hypertrophic cardiomyopathy clinic: the HADS in a cardiomyopathy population. *Gen Hosp Psychiatry*. 2006;28(1):55-58. doi:10.1016/J.GENHOSPPSYCH.2005.08.004
8. Christensen AV, Dixon JK, Juel K, et al. Psychometric properties of the Danish Hospital Anxiety and Depression Scale in patients with cardiac disease: results from the DenHeart survey. *Health Qual Life Outcomes*. 2020;18(1). doi:10.1186/S12955-019-1264-0
9. Bambauer KZ, Locke SE, Aupont O, Mullan MG, McLaughlin TJ. Using the Hospital Anxiety and Depression Scale to screen for depression in cardiac patients. *Gen Hosp Psychiatry*. 2005;27(4):275-284. doi:10.1016/J.GENHOSPPSYCH.2005.03.002
10. Eifert GH, Thompson RN, Zvolensky MJ, et al. The cardiac anxiety questionnaire: development and preliminary validity. *Behaviour research and therapy*. 2000;38(10):1039-1053. doi:10.1016/S0005-7967(99)00132-1
11. Mourad G, Strömberg A, Johansson P, Jaarsma T. Depressive Symptoms, Cardiac Anxiety, and Fear of Body Sensations in Patients with Non-Cardiac Chest Pain, and Their Relation to Healthcare-Seeking Behavior: A Cross-Sectional Study. *Patient*. 2016;9(1):69-77. doi:10.1007/S40271-015-0125-0
12. Sardinha A, Nardi AE, de Araújo CGS, Ferreira MC, Eifert GH. Brazilian Portuguese Validated Version of the Cardiac Anxiety Questionnaire. *Arq Bras Cardiol*. 2013;101(6):554. doi:10.5935/ABC.20130207
13. Van Beek M, Voshaar R, Van Deelen F, Van Balkom A, Pop G, Speckens A. The cardiac anxiety questionnaire: cross-validation among cardiac inpatients. *Int J Psychiatry Med*. 2012;43(4):349-364. doi:10.2190/PM.43.4.E
14. Dragioti E, Vitoratou S, Kaltsouda A, Tsartsalis D, Gouva M. Psychometric properties and factor structure of the Greek version of the Cardiac Anxiety Questionnaire (CAQ). *Psychol Rep*. 2011;109(1):77-92. doi:10.2466/08.09.15.PR0.109.4.77-92

15. Rosman L, Whited A, Lampert R, Mosesso VN, Lawless C, Sears SF. Cardiac anxiety after sudden cardiac arrest: Severity, predictors and clinical implications. *Int J Cardiol*. 2015;181:73-76. doi:10.1016/J.IJCARD.2014.11.115
16. Zuchowski M, Chilcot J. Illness Perceptions in Hypertrophic Cardiomyopathy (HCM) Patients and Their Association With Heart-Focussed Anxiety. *Heart Lung Circ*. 2021;30(4):496-506. doi:10.1016/J.HLC.2020.08.009
17. Spertus JA, Jones PG, Sandhu AT, Arnold S V. Interpreting the Kansas City Cardiomyopathy Questionnaire in Clinical Trials and Clinical Care: JACC State-of-the-Art Review. *J Am Coll Cardiol*. 2020;76(20):2379-2390. doi:10.1016/J.JACC.2020.09.542
18. Green CP, Porter CB, Bresnahan DR, Spertus JA. Development and evaluation of the Kansas City Cardiomyopathy Questionnaire: a new health status measure for heart failure. *J Am Coll Cardiol*. 2000;35(5):1245-1255. doi:10.1016/S0735-1097(00)00531-3
19. Spertus JA, Fine JT, Elliott P, et al. Mavacamten for treatment of symptomatic obstructive hypertrophic cardiomyopathy (EXPLORER-HCM): health status analysis of a randomised, double-blind, placebo-controlled, phase 3 trial. *Lancet*. 2021;397(10293):2467-2475. doi:10.1016/S0140-6736(21)00763-7
20. Olivotto I, Oreziak A, Barriales-Villa R, et al. Mavacamten for treatment of symptomatic obstructive hypertrophic cardiomyopathy (EXPLORER-HCM): a randomised, double-blind, placebo-controlled, phase 3 trial. *Lancet*. 2020;396(10253):759-769. doi:10.1016/S0140-6736(20)31792-X
21. Johansson I, Joseph P, Balasubramanian K, et al. Health-Related Quality of Life and Mortality in Heart Failure: The Global Congestive Heart Failure Study of 23 000 Patients From 40 Countries. *Circulation*. 2021;143(22):2129-2142. doi:10.1161/CIRCULATIONAHA.120.050850
22. Spertus JA, Jones PG. Development and Validation of a Short Version of the Kansas City Cardiomyopathy Questionnaire. *Circ Cardiovasc Qual Outcomes*. 2015;8(5):469-476. doi:10.1161/CIRCOUTCOMES.115.001958
